# Murine Regulatory CD4^+^ T Cells Are Increased in Leukemic Spleens and Show High Co-Expression of Co-Regulatory Molecules CD39, CD73, PD1, and TIGIT

**DOI:** 10.3390/ijms252111412

**Published:** 2024-10-24

**Authors:** Julius Krüger, Jasmin Wellbrock, Marius Witt, Niklas Kruppa, Jana Muschhammer, Carsten Bokemeyer, Franziska Modemann, Walter Fiedler, Lena Behrmann, Franziska Brauneck

**Affiliations:** Department of Oncology, Hematology and Bone Marrow Transplantation with Section Pneumology, Hubertus Wald University Cancer Center, University Medical Center Hamburg-Eppendorf, 20251 Hamburg, Germany; julius.krueger@stud.uke.uni-hamburg.de (J.K.); marius.witt@stud.uke.uni-hamburg.de (M.W.); niklas.kruppa@stud.uke.uni-hamburg.de (N.K.); j.muschhammer@uke.de (J.M.); c.bokemeyer@uke.de (C.B.); f.modemann@uke.de (F.M.); le.behrmann@uke.de (L.B.); f.brauneck@uke.de (F.B.)

**Keywords:** murine AML, spleen, T cells, regulatory CD4^+^ T cells, antitumor immunity, checkpoint molecules, TIGIT, PD-1, CD39, CD73

## Abstract

Comprehensive characterization of AML-associated T cells during disease progression is essential to identify relevant immune escape mechanisms and new immunotherapeutic approaches. Investigating the processes that lead to an immunosuppressive environment under progression of AML is difficult in humans, because by the time of diagnosis the disease is often progressed far beyond the initial stages. Therefore, to investigate T-cell phenotypes during progression a C57BL/6 mouse model was used. The CD3^+^ T cells were characterized by performing multiparametric flow analyses at different time points (day 0 = healthy mice, day 7, day 14, and day 21). The study revealed that the spleen is highly infiltrated by reg CD4^+^ T cells at day 21 of AML progression. These spleen-infiltrating reg CD4^+^ T cells mainly showed an effector memory differentiation with high expression and co-expression of the checkpoint molecules TIGIT, PD-1, OX40, and the two ectoenzymes CD39 and CD73. Substantial expression of the checkpoint molecules was restricted to the central memory and effector memory compartments. Furthermore, functional evaluation of TIGIT was performed. Blocking TIGIT resulted in a significantly increased lysis of C1498 AML cells in cocultures with AML-primed CD3^+^ T cells. Together these data confirm that the expression of the checkpoint receptor TIGIT is relevant for dysfunction of AML-associated T cells and, thus, represents a suitable target for future immunotherapeutic approaches.

## 1. Introduction

Acute myeloid leukemia (AML) is a heterogeneous neoplasm, as evidenced by its clinical, cytogenetic, and molecular characteristics [1]. New immunotherapeutic strategies aimed at improving T-cell function are constantly being developed for the treatment of AML. These include vaccines, T-cell-recruiting bispecific antibody constructs, chimeric antigen receptor (CAR) T cells and immune checkpoint inhibitors [2]. However, apart from allogeneic stem cell transplantation, the clinical success of such novel T-cell-based strategies has remained limited compared to other tumor entities [3,4]. One reason for this is certainly the heterogeneous nature of the disease whereby different immunosuppressive mechanisms must be treated to overcome the antileukemic immunosuppression.

To develop potent new immune strategies—probably also multispecific ones—detailed knowledge of T-cells’ function and their manifold depletion over the course of the disease is essential. This is not yet sufficiently understood.

In chronic infections and cancer, T cells are exposed to sustained antigen stimulation, often associated with loss of T-cell function and upregulation of multiple inhibitory receptors [5]. Among them, inhibitory receptors and their ligands, such as the programmed cell death protein 1/programmed cell death ligand 1 (PD-1/PD-L1), play a crucial role in the regulation of inflammatory responses by inhibiting the effector activity of T cells [6]. We and others have recently identified the axis of the co-inhibitory receptor T-cell immunoglobulin and immunoreceptor tyrosine-based inhibitory motif (ITIM) domain (TIGIT) as a potential pathway for immunotherapeutic strategies in human AML [7,8]. TIGIT is expressed on (virus-specific) CD8^+^ and conventional CD4^+^ (con CD4^+^), as well as regulatory CD4^+^ (reg CD4^+^) T cells, under sustained T-cell receptor stimulation [7].

On the other hand, costimulatory receptors such as OX40 (CD134) are expressed because of T-cell activation and mediate the survival and expansion of both CD4^+^ and CD8^+^ T cells in several animal models, which also plays an important role in autoimmunity, infectious diseases, and cancer [9]. OX40 is also involved in the control of effector and memory T-cell responses [10].

In addition, ectonucleoside triphosphate diphosphohydrolase 1 (CD39) and ecto-5’-nucleotidase (CD73) have recently been described in the context of T-cell exhaustion, as they are involved in purinergic signaling, which converts adenosine triphosphate (ATP) released by cells under stress or inflammatory conditions or by regulated secretion into adenosine (ADO) by sequential dephosphorylation [11]. ADO triggers preemptive anti-inflammatory signaling [12]. In addition to its function in ADP degradation, CD73 appears to play a role as a costimulatory molecule for T-cell differentiation [12,13]. In contrast, expression of CD39 was found on antigen-specific (tumor-infiltrating) CD8^+^ T cells, con CD4^+^, and reg CD4^+^ T cells [12,13].

The phenotypical characterization of T cells during AML progression involving the abovementioned co-regulatory molecules is still lacking. Since this is not yet possible in human models, we have chosen a syngeneic C57BL/6 mouse model in which we characterized T-cell infiltration, differentiation, and expression of co-regulatory molecules at different time points. Since our preliminary data showed that T cells in C57 BL/6 mice infiltrate mainly the spleen, the following phenotypic characterization is focused on spleen infiltrating T cells under AML progression.

## 2. Results

### 2.1. TIGIT, PD-1, OX40, CD39, and CD73 Are Expressed on Murine Reg CD4^+^ T Cells

The T-cell destruction in the context of chronic T-cell receptor (TCR) stimulation is one of the most important mechanisms responsible for the immunological escape of tumor cells. This has already been shown for human T cells [14]. Investigating tumor progression in a mouse model offers the unique opportunity to study the development of T cells under tumor progression in a uniform setting. In a syngeneic mouse model, we injected C1498 AML cells into 12- to 15-week-old C57BL/6 mice. After 7, 14, and 21 days, the mice were sacrificed (d0, n = 4; d7, n = 4; d14, n = 4; and d21, n = 6), and mononuclear cells were isolated from the spleen to investigate the T-cell status using multiparametric flow cytometry (MFC). Since the cell line C1498 induces a very aggressive AML, our last time point represents a very advanced stage of the disease [15]. Please refer to Supplemental Figure S1A for a survival curve of n = 7 BL/6 mice after injection of 10^6^ C1498. We decided to focus on splenic cells as the frequency of CD3^+^ T cells is low in the bone marrow of C57BL/6 mice, as observed in our own prior experiments and studies published by others (Supplementary Figure S1B,C) [16].

**Figure 1:**
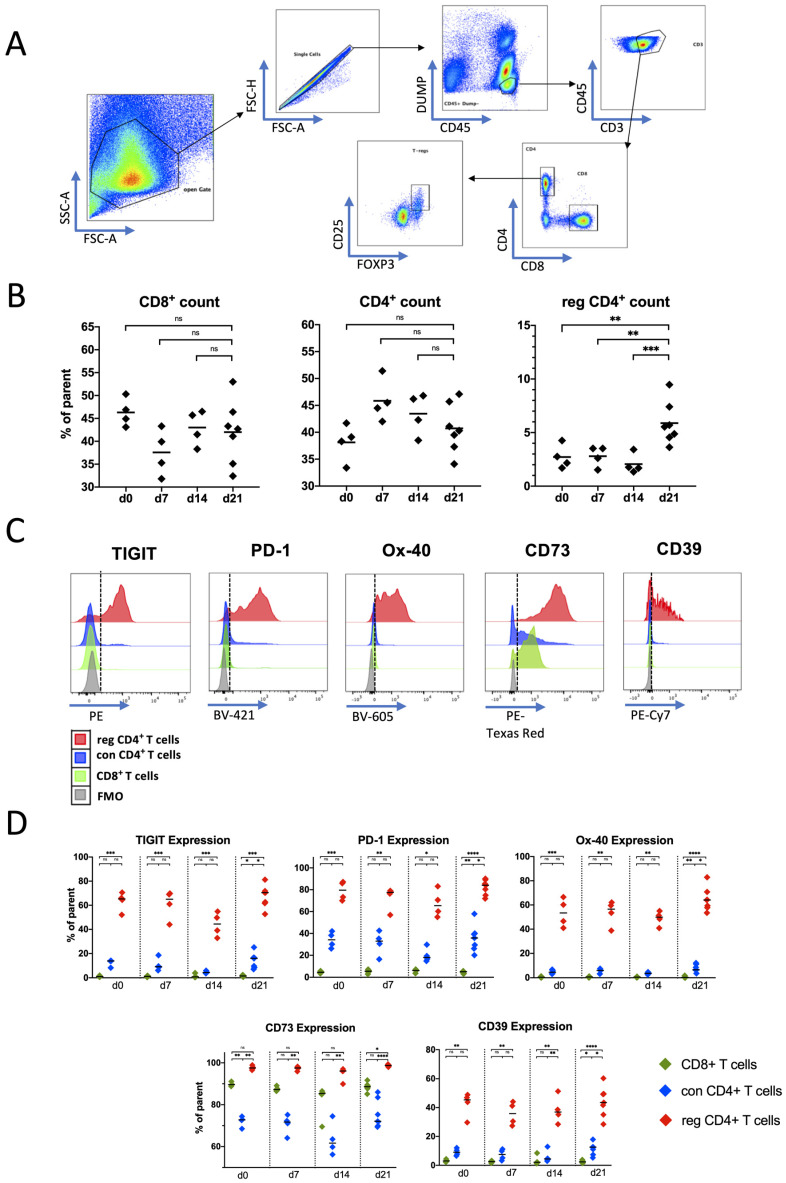
Multiparametric flow cytometry (MFC) of the spleen-derived CD3^+^ T cells was performed on splenocytes from healthy mice (d0, n = 4) and leukemic mice at different time points after transplantation (d7, n = 4; d14, n = 4; and d21, n = 6): (**A**) exemplary gating strategy on FACS plots; (**B**) frequencies of CD4^+^, CD8^+^, and reg CD4^+^ T cells at different time points in relation to their parent population (CD3^+^ cells for CD4^+^ and CD8^+^ T cells; CD4^+^ T cells for reg CD4^+^ T cells); (**C**) exemplary illustration of the fluorescence intensity for the different immune checkpoint (IC) molecules against fluorescence minus one control (FMO). The vertical line indicates fluorescence level of FMO; (**D**) summary data of IC expression on CD4^+^ T cells, CD8^+^ T cells, and reg CD4^+^ T cells. Frequencies are displayed with the median. *p*-values were obtained by the Kruskal–Wallis test or the ordinary one-way ANOVA. * *p* < 0.05, ** *p* < 0.01, *** *p* < 0.001, **** *p* < 0.0001 and ns = not significant.

A representative MFC gating strategy is illustrated in Figure 1A. The spleen infiltration by CD8^+^ and con CD4^+^ T cells was constant across all four time points of analysis, whereas a significantly increased frequency of reg CD4^+^ T cells was detected on day 21 in comparison to the other days (d21 vs. d0: *p* < 0.01; d21 vs. d7: *p* < 0.01; d21 vs. d14: *p* < 0.001; Figure 1B).

For human models, it has already been shown that T cells exhibit altered expression of their co-regulatory receptors under tumor progression due to prolonged TCR stimulation [14]. This mechanism has been described as T-cell exhaustion. Built on these data, we investigated the expression of co-regulatory receptors during AML progression in our mouse model. The present study includes expression analyses of the coinhibitory receptors TIGIT, PD-1, Tim-3, and Lag-3; the costimulatory receptor OX40; and the two ectoenzymes CD73 and CD39.

When analyzing the expression of immune checkpoint markers on different T-cell subsets, we observed that TIGIT, PD1, OX40, CD39, and CD73 emerged as checkpoints of interest. Lag-3 and Tim-3 showed very low and inconsistent expression over all types of splenic CD3^+^ cells (Supplementary Figure S2A,B). There was an increased frequency of TIGIT^+^, PD-1^+^, and OX40^+^ reg CD4^+^ T cells, whereas their corresponding CD8^+^ or con CD4^+^ T cells displayed rather low expression of these markers (Figure 1C,D). The expression pattern of the ectoenzymes CD39 and CD73 also differed among corresponding CD8^+^/con CD4^+^/reg CD4^+^ T cells. CD39 was significantly more frequent on reg CD4^+^ T cells than on CD8^+^ or con CD4^+^ T cells in spleens, at all analyzed time points. The expression of CD73 was high in all analyzed T-cell subsets. Reg CD4^+^ T cells had the highest expression, followed by CD8^+^ T cells. CD73 showed the lowest expression on con CD4^+^ T cells, although it was still expressed on the majority of con CD4^+^ T cells. (Figure 1C,D).

We also analyzed median fluorescence intensity (MFI) of the co-regulatory molecules’ expression, which showed similar results (Supplemental Figure S3A).

Together, the study did not detect significant differences in CD8^+^ and con CD4^+^ T-cell infiltration of spleens at different time points. Reg CD4^+^ T cells appeared to be the most interesting population, as we found a significantly increased fraction of these cells in the spleens at our latest time point after leukemia transplantation and highest frequencies of immune checkpoint receptor expressing cells within this T-cell population.

### 2.2. Murine EM and CM Reg CD4^+^ T Cells More Frequently Express TIGIT, PD-1, OX40, CD39 and CD73

**Figure 2:**
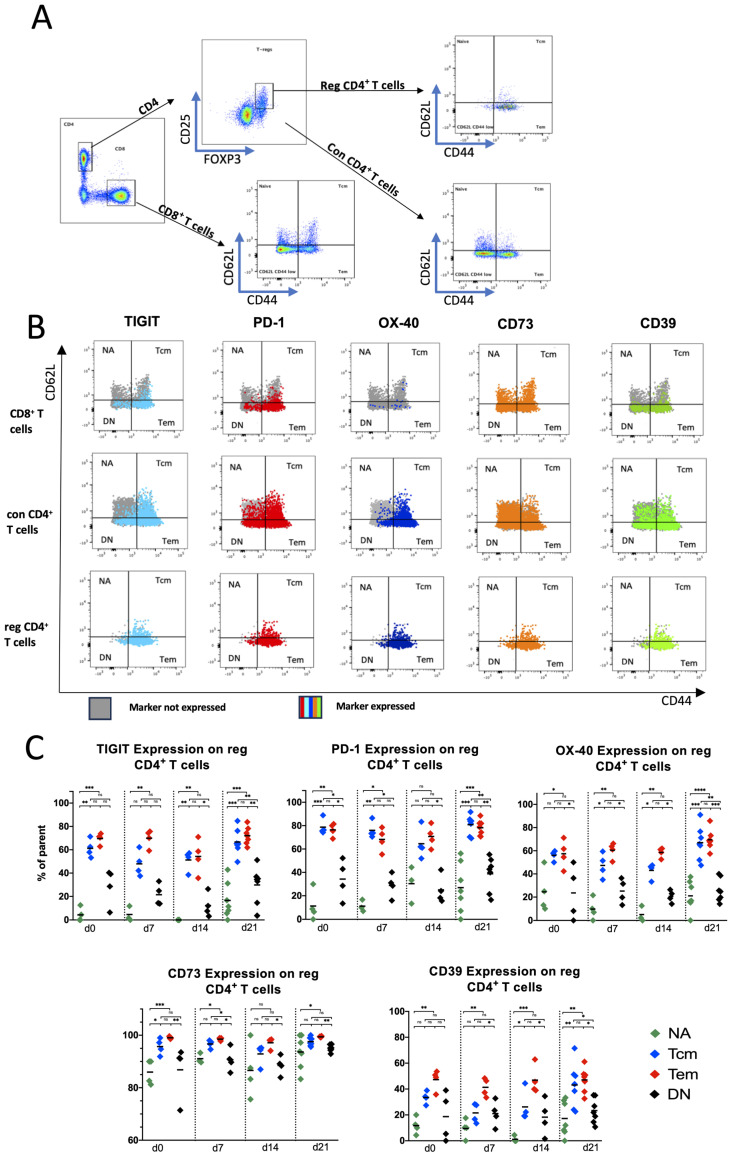
(**A**) Exemplary gating strategy for the T-cell subgroups NA = CD62L^+^CD44^−^; Tem = CD62L^−^CD44^+^; Tcm = CD62L^+^CD44^+^; and DN (double-negative) = CD62L^−^CD44^−^ subpopulations; (**B**) exemplary distribution patterns of the immune checkpoint expression on the different T-cell subpopulations; (**C**) summary data of immune checkpoint expression on T-cell subgroups. Frequencies are displayed with the median. *p*-values were obtained by the Kruskal–Wallis test. * *p* < 0.05, ** *p* < 0.01, *** *p* < 0.001, **** *p* < 0.0001 and ns = not significant.

To further investigate whether the expression of co-regulatory molecules is related to T-cell differentiation, we examined the differentiation status of CD8^+^ T cells, con CD4^+^ T cells, and reg CD4^+^ T cells. Here, we defined NA (naive) = CD62L^+^CD44^−^; Tem (effector memory) = CD62L^−^CD44^+^; Tcm (central memory) = CD62L^+^CD44^+^; and DN (double-negative) = CD62L^−^CD44^−^ subpopulations (an exemplary gating strategy is illustrated in Figure 2A).

There were no significant changes regarding the differentiation status of T cells between nonleukemic mice (d0) and leukemic mice (d7, d14, and d21). However, there were differences among the T-cell subpopulations that were independent of the time of analysis. In CD8^+^ T cells, DN was the most frequent phenotype, followed by Tem. In the conventional CD4^+^ T cells, Tems and DN represented the most prominent cell fractions, while in reg CD4^+^ T cells, the Tem population represented the most dominant differentiation status. In general, the fractions of NA and Tcm cells was low among CD8^+^, con CD4^+^, and reg CD4^+^ T cells (Supplemental Figure S4A).

We then compared the expression of TIGIT, PD-1, OX40, CD39, and CD73 among the individual differentiation stages of the T cells. Here, we focused our analyses on the reg CD4^+^ T cells based on the finding that they largely expressed the immune checkpoint receptors. As shown in Figure 2B, the reg CD4^+^ EM and CM populations also proved to be the most interesting, as TIGIT, PD-1, OX40, CD39, and CD73 were expressed significantly more frequently in both subpopulations than in the NA or DN subpopulation (Figure 2C). We also found the same patterns, albeit on a lower base expression level, for CD8^+^ and con CD4^+^ T cells (Supplemental Figure S4B,C).

In summary, our analyses of splenic infiltrating T cells in the murine model confirmed what has already been observed for human bone-marrow-infiltrating T cells, that the immune checkpoint receptors are mainly expressed by CM and EM subpopulations. As aforementioned, we further confirmed these expression results also via the MFI (Supplemental Figure S3B,C).

### 2.3. CD39 and CD73 Are Co-Expressed on TIGIT^+^PD1^+^ Reg CD4^+^ T Cells

**Figure 3:**
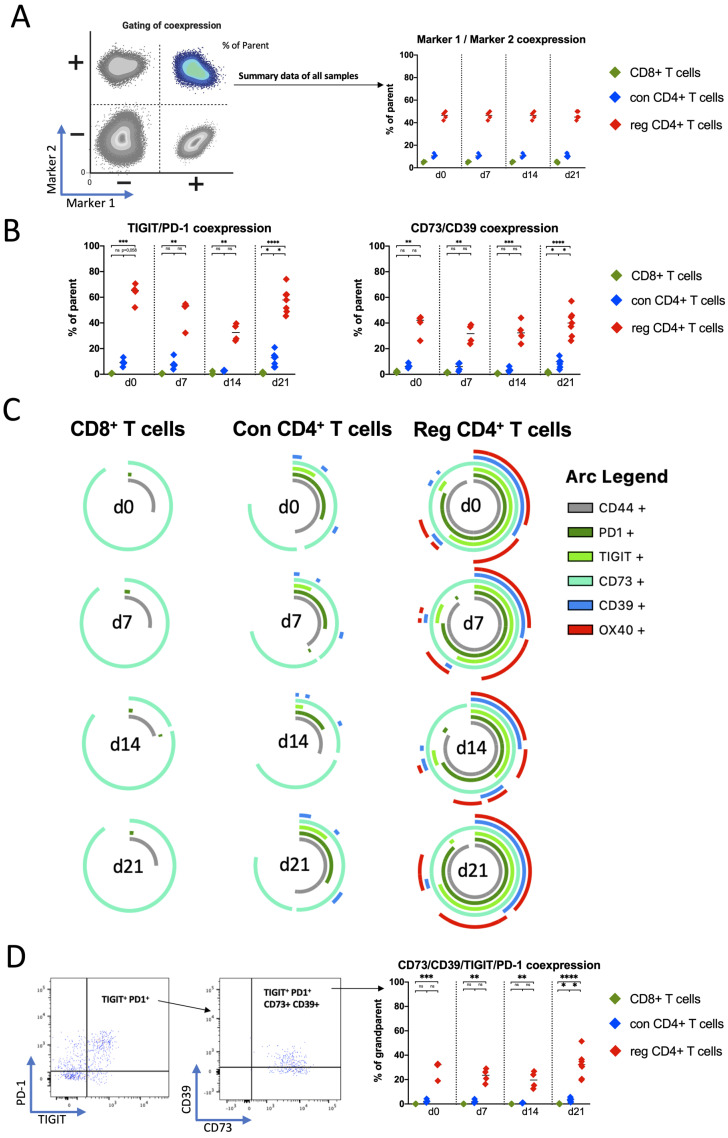
(**A**) Exemplary gating strategy for the co-expression data; (**B**) summary data of TIGIT/PD-1 and CD39/CD73 co-expression; (**C**) SPICE analysis of five co-regulatory receptors at different time points and on different T-cell subsets, and the overlapping arcs illustrate co-expression for these molecules; (**D**) gating strategy and summary data of the quadruple expression of TIGIT/PD-1/CD39/CD73. Frequencies are displayed with the median. *p*-values were obtained by the Kruskal–Wallis test. * *p* < 0.05, ** *p* < 0.01, *** *p* < 0.001, **** *p* < 0.0001 and ns = not significant.

Previous findings have shown that PD-1-positive/TIM3-positive (double-positive) T cells in mouse models of AML represent highly exhausted T cells within the immune microenvironment and are associated with earlier relapse after ASCT in patients with AML [17]. Furthermore, aberrant co-expression of TIGIT with PD-1 or CD39 on CD8^+^ T cells has been previously described for patients with AML [18]. We examined the frequency of each T-cell subset co-expressing TIGIT, PD-1, CD39, and CD73 or even quadruple expression in spleens of our murine C57BL/6 mice with AML (illustrated in Figure 3A).

Comparing TIGIT/PD-1 co-expression gated on each T-cell subset, an increased frequency of TIGIT^+^PD-1^+^ on reg CD4^+^ T cells in spleens at every time point of analysis was observed (at day 21 post-transplantation: reg CD4 vs. CD8 *p* < 0.0001 and reg CD4 vs. con CD4 *p* = 0.047; Figure 3B). In addition, also CD39 and CD73 were more frequently co-expressed by reg CD4^+^ T cells in spleens than on their corresponding CD8^+^ or con CD4^+^ T cells (Figure 3B).

For a better overview of all co-expressed molecules, we performed a simplified presentation of incredibly complex evaluations (SPICE) analysis, including TIGIT, PD-1, OX-40, CD39, and CD73 [19]. The SPICE analysis displayed a high fraction of reg CD4^+^ T cells expressing multiple co-regulatory molecules at once, while CD8^+^ T cells and con CD4^+^ T cells did not nearly show this to the same extent, confirming our previous findings in this study. To quantify the co-expression pattern seen in the SPICE diagrams, we compared the fraction of cells expressing all four—TIGIT, PD-1, CD39, and CD73—among the T-cell subpopulations. Again, reg CD4^+^ T cells displayed a significantly higher fraction of CD39^+^CD73^+^ cells within the subpopulation of TIGIT^+^PD-1^+^ cells than CD8^+^ T cells or con CD4^+^ T cells maximally pronounced at day 21 post-transplantation (*p* < 0.0001 and *p* = 0.0378; Figure 3D).

Together, the reg CD4^+^ T cells showed an increased frequency of cells simultaneously expressing multiple co-regulatory receptors at all time points. At the last analysis time point on day 21, the co-expression patterns were most frequent.

This finding suggests that T cells in contact with AML cells have an aberrant profile of immunologically relevant receptors that may influence T-cell function.

### 2.4. Blockade of TIGIT Increased Lysis of AML Cells in Cocultures with AML Primed T Cells Ex Vivo

**Figure 4:**
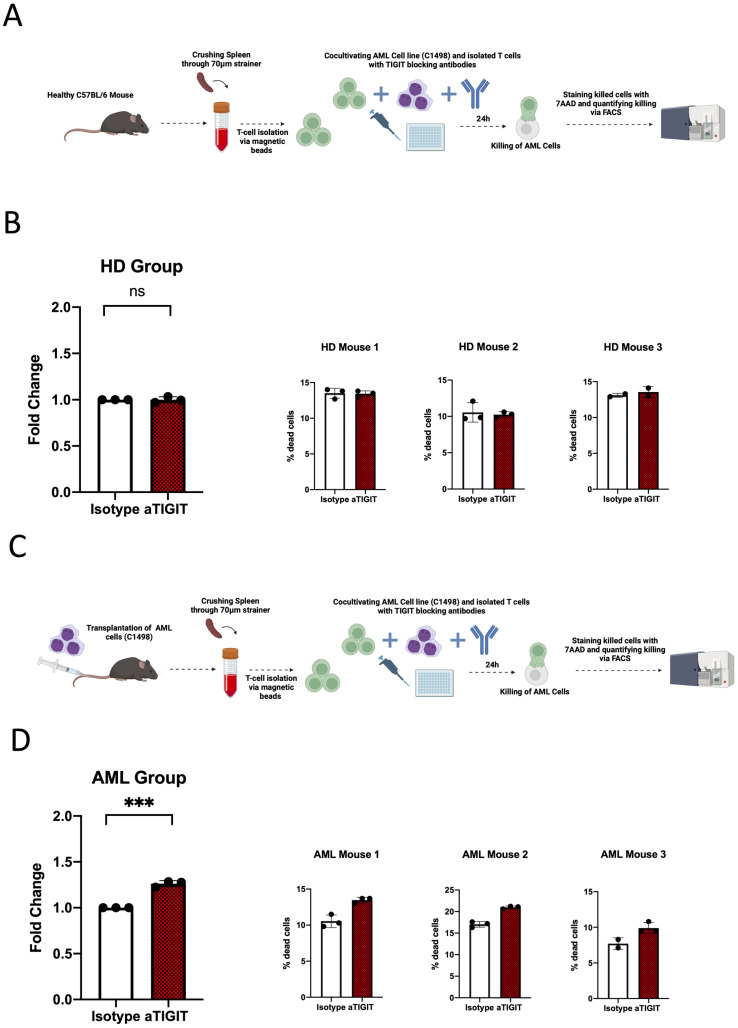
(**A**) Illustration of ex vivo cytotoxicity assay methodology in the healthy donor (HD) cohort; (**B**) quantity of dead tumor cells compared between the isotype and aTIGIT groups in the HD cohort; (**C**) illustration of ex vivo cytotoxicity assay methodology in the leukemia cohort; (**D**) quantity of dead tumor cells compared between the isotype and aTIGIT groups in the leukemia cohort. Frequencies are displayed with the median. *p*-values were obtained by the unpaired *t* test. *** *p* < 0.001 and ns = not significant. Illustrations were created with BioRender.com.

Finally, our aim was to identify new target structures for immunotherapeutic strategies in AML that we could also further investigate regarding their functional relevance. Because of the frequent expression of TIGIT, we decided to focus on its functional evaluation. Again, we used the syngeneic mouse model where we transplanted C1498 AML cells into C57BL/6 mice. At day 14, mice were sacrificed, and T cells were isolated from the spleen. The T cells and C1498 AML were subsequently cocultured for ex vivo in cytotoxicity assays (Figure 4A). Cocultures were treated with TIGIT-blocking antibodies (anti-mouse TIGIT clone F132-1B4, kindly provided by Prof. Vijay Kuchroo, Harvard Medical School) or respective isotype controls (mouse IgG1k). At day 0, the high expression of TIGIT was found on spleen T cells. After 24 h of coculturing and treatment, an evaluation of the lysed C1498 cells was performed using 7AAD staining for quantification of the percentage of lysis via MFC. Blockade of TIGIT in coculture with T cells from nonleukemic mice cells showed no significant difference in C1498 lysis (*p* = 0.99; Figure 4B). In contrast, blockade of TIGIT in cocultures containing previously AML-primed T cells (obtained from the C57BL/6 mice) and C1498 AML cells showed a significantly increased lysis of C1498 cells in comparison to the controls (*p* < 0.01; Figure 4D).

The TIGIT blockade affected T-cell function in T cells that had previously undergone priming in contact with AML cells. Blockade of this checkpoint receptor augmented the T-cell-mediated lysis of AML cells.

## 3. Discussion

The study showed in a murine C57BL/6 model that the spleen is primarily infiltrated by reg CD4^+^ T cells during the progression of AML. CD3^+^ T cells isolated from the spleen expressed immune checkpoint receptors such as TIGIT, PD-1, OX40, as well as the two ectoenzymes CD39 and CD73, on their surface more often. The expression frequencies were found to be independent of the time of measurement/AML progression for each cell type and subgroup. Interestingly, within the different T-cell populations, all target molecules were found more frequently on reg CD4^+^ T cells. In our analysis, these reg CD4^+^ T cells highly infiltrated the spleen at the late time points after leukemia transplantation, thereby exposing the immune system of leukemic mice to immunoregulatory molecules. These findings suggest, that reg CD4^+^ T cells are an important contributor to the immunosuppressive environment in the late stages of AML.

The T-cell differentiation studies showed that that the proportion of DN CD8+ T cells was high in all analyzed groups including the nonleukemic control mice, as well the AML-mice at all analyzed time points. Interestingly, Nakajima et al. described that this subpopulation differentiates from naive T cells into effector T cells after tumor cell inoculation and represents a kind of pre-effector-like T cell [20]. However, only a few studies on this subpopulation have been published, and further analysis is needed to elucidate its function in the antitumor response. With regard to their differentiation reg CD4^+^ T-cells infiltrating, the spleen showed significantly fewer cells of NA, CM, and DN differentiation, compared to the EM phenotype.

Interestingly, TIGIT, PD-1, OX40, CD39, and CD73 were mainly expressed by EM and CM T cells.

According to current studies, co-expression of several immune checkpoint receptors is considered a sign of dysfunctional “exhausted” T cells. In our study, CD3^+^ T cells from the spleen co-expressed TIGIT and PD-1, as well as CD39 and CD73, particularly frequently. Again, compared to the CD8^+^ and con CD4^+^ T cells, we found reg CD4^+^ T cells co-expressing CD39 and CD73 on TIGIT^+^PD1^+^ cells statistically more frequently. In our human models, we already showed a co-expression of TIGIT with CD39 or PD-1 on CD3^+^ cells but did not see co-expression of CD39 and CD73 [18].

Since TIGIT was expressed more frequently, we decided to further investigate the functional relevance of TIGIT. The effect on T-cell-mediated lysis of AML cells was investigated in different cocultures. The blockade of TIGIT resulted in an increased lysis of AML cells in cocultures with AML-primed T cells. These initial functional studies further confirmed that the expression of the checkpoint receptor TIGIT is relevant for AML-associated T cells and could be a suitable target for future immunotherapeutic approaches.

In a previous AML mouse model, it was shown that the spleen reacts to the progression of transplanted C1498 leukemia [21]. However, in this study, mainly cells from the PB or BM were examined in detail for their T-cell and tumor infiltration. It was shown that immune cell infiltration of PB and BM occurs in response to the transplanted leukemia.

Our investigations have confirmed that the spleen is a T-cell-rich organ, which has enabled us to obtain large quantities of cells for our expression studies and functional experiments [22].

The substantial infiltration with reg CD4^+^ T cells at late stages of leukemia was an interesting finding. This infiltration with reg CD4^+^ T cells has already been observed in humans, in which blood from leukemia patients contained significantly more reg CD4^+^ T cells than HD samples [23]. Moreover, high infiltration of reg CD4^+^ T cells could be associated with poor prognosis [24]. In addition, multiple studies showed that reg CD4^+^ T cells contribute to leukemia progression by establishing an immune-privileged niche benefiting the proliferation of leukemia stem cells (LSCs) by protecting them from elimination via CD8^+^ T cells [25,26].

Various studies could previously show that immune checkpoints play an important role in the immune response to AML.

For example, for the co-inhibitory receptor TIGIT, human studies identified that TIGIT exerts immunomodulatory functions in the context of chronic infections [27]. Ackermann et al. showed that virus-specific T cells with an increased expression of TIGIT displayed a lower cytotoxic potential [28]. In addition, TIGIT has also been described on exhausted T cells in tumor diseases, especially in the context of its involvement in various immunosuppressive mechanisms like promoting dysfunctional phenotypes in macrophages or NK cells [29,30]. Our group was among the first to reveal the therapeutic potential of the TIGIT axis. Blocking TIGIT or its ligands PVR or PVRL2 increased the efficacy of immune effector cells in vitro. Furthermore, in a humanized mouse model, mice transplanted with a human PVR/PVRL2 double-knockout AML cell line survived longer than the control group being inoculated with the parental cell line with high PVR and PVRL2 expression [31]. By confirming these findings in our ex vivo model with AML-primed T cells, we have discovered an additional investigation model for further research.

In line with these data, improved antitumor immunity by blockade of TIGIT was also found for solid tumor models including colon-cancer-bearing mice [32]. Fuhrman et al. observed that TIGIT is highly expressed and upregulated on regulatory T cells after activation and in vitro expansion and that it is, furthermore, associated with lineage stability and suppressive capacity [33]. Furthermore, using mouse models, Joller et al. identified TIGIT^+^Foxp3^+^ T cells as a distinct T-reg-cell subset that specifically suppresses pro-inflammatory Th1 and Th17 cells but not Th2 cell responses. TIGIT^+^ T regs produced higher amounts of effector molecules, such as IL-10, Fgl2, and granzyme B, compared to TIGIT^−^ T regs. The production of both IL-10 and Fgl2 by T regs could be induced using an agonistic TIGIT antibody and neutralization of Fgl2 reversed the enhanced suppressive capacity of TIGIT^+^ Tregs in vitro. TIGIT^+^ Treg cells, therefore, represent more potent suppressors than TIGIT^−^ T regs [34]. The PD-1^−/−^ C57BL/6 mice showed significantly prolonged overall survival and tumor antigen-specific T-cell priming [35]. That PD-1 could play an important role in AML has already been shown in human studies, the expression of PD-1, especially co-expression with other checkpoint molecules, was associated with poor prognosis [36]. Tan et al. generated mice that selectively lack PD-1 in T regs. The PD-1-deficient T regs exhibited an activated phenotype and enhanced immunosuppressive function in autoimmune disease [37]. In line with this, Lowther et al. have shown that PD-1^high^ T regs display reduced suppression of CD4^+^ effector T cells, production of IFN-γ, and molecular signatures of exhaustion [38]. In the clinical setting, the blockade of PD-1 resulted in increased proliferation and enhanced suppressive function of tumor-infiltrating T regs resulting in rapid tumor progression in 10% of advanced gastric cancer patients [39]. These data show that PD-1 blockade can have both anti- and pro-tumor effects, depending on the patients’ immune cell composition.

Furthermore, the co-stimulatory receptor OX40, whose expression was more frequently found on our EM and CM cells, has already been identified as a prognosis-relevant factor and potential target of immunotherapy in human studies [40]. OX40 is a co-stimulatory molecule that is expressed in the late activation phase of T cells [41]. In an autoimmune ovalbumin (OVA)-induced mouse asthma model, OX40L-treated and OX40^+^ T cells in mice showed enhanced asthma development through OX40/OX40L signaling. This was likely mediated by increased expression of inflammatory factors, infiltration of eosinophils, and proliferation of T cells [42]. The increased expression on T cells in our murine C57BL/6 model suggests that through AML progression the T cells were initially activated. For human T cells derived from AML patients, it was recently demonstrated that stimulation by OX40 leads to enhanced T-cell proliferation and expansion. Re-activation of exhausted T cells in patients in remission was also characterized by activation of the OX40 pathway, highlighting the importance of the interplay between co-inhibitory and co-stimulatory pathways in defining T-cell functionality in the context of active disease and after treatment [43].

We also investigated the expression levels and patterns of the purinergic signaling molecules CD73 and CD39. Especially on CD8^+^ T-cell and reg CD4^+^ T-cell fractions, we found a high expression of these molecules, with CD73 and CD39 also being co-expressed by a significant fraction of reg CD4^+^ T cells. Furthermore, it was shown that CD39 expression is involved in T reg stability [44]. Recent studies also suggest the purinergic signaling pathway to be an interesting target for immunotherapy in AML and other malignancies [45]. In melanoma, low levels of CD39^+^CD25^+^ T regs were associated with improved relapse-free survival [46].

Co-expression of multiple co-inhibitory receptors has been found on T cells in other tumor entities, such as glioblastoma, and indicates a high degree of T-cell exhaustion [47,48,49]. In our study, co-expression of multiple checkpoint molecules mainly occurred in reg CD4^+^ T cells, which also showed an overall high expression of immune checkpoint molecules. Other studies also describe the occurrence of immune checkpoint molecule co-expression on reg CD4^+^ T cells in various models [50,51]. Further research is needed to decipher the underlying mechanisms of T reg biology in cancer progression. These studies should shed light on which immune checkpoint blockade could be the most successful to reduce the immunosuppressive effects of T regs.

## 4. Materials and Methods

### 4.1. Collecting Splenocytes from Leukemic Mice

Splenocytes were collected from C57 BL/6 mice, who had been transplanted with C1498 AML cells (n = 22), as well as from healthy mice (n = 8). The leukemic mice were killed at various points in time after transplantation with n = 4 after 7 days, n = 8 after 14 days, and n = 10 after 21 days, to examine differences over the course of the disease. The mice were killed by a lethal dose of ketamine and xylazine, followed by cervical dislocation. The spleen was then removed, crushed through a 20 μm cell strainer, and washed with PBS. After that, we performed erylysis. The cells were then counted and cryopreserved. All animal experimental procedures in this study comply with the German Animal Welfare Act and the European Guideline EU 2010/63 and have been approved by the local authorities.

### 4.2. Multiparameter Flow Cytometry and Surface Staining

For the multiparameter flow cytometry (MFC) analyses, cryopreserved splenic cells were thawed in a 37 °C water bath and counted. For a high-quality analysis, samples with a viability of under 70% after thawing were not used for MFC staining; therefore, the final number of samples was n = 18 (d0, n = 4; d7, n = 4; d14, n = 4; d21, n = 6).

Then, 3 × 10^6^ cells from each sample were washed with PBS (phosphate-buffered saline) (Dulbecco’s Phosphate Buffered Saline, Gibco Thermo Fisher Scientific, Waltham, MA, USA). After FcR blocking (FcR Blocking Reagent, mouse, Miltenyi Biotec, Bergisch Gladbach, Germany) for 5 min in the dark, the cells were stained with the Zombie NIR^™^ Fixable Viability Kit (BioLegend, San Diego, CA, USA) for exclusion of dead cells according to the manufacturer’s instructions. This step was followed by another washing step with PBS. For surface staining, cells were incubated with appropriate fluorochrome-conjugated antibodies, including anti-CD3 (17A2), anti-CD4 (GK1.5) anti-CD8 (53-6.7), anti-CD45 (RAT LOU), anti-CD44 (IM7), anti-CD62L (MEL-14), anti-CD25 (3C7), anti-TIGIT (A15153G), anti-PD-1 (29F.1A12), anti-LAG-3 (C9B7W), anti-CD39 (Duha59), anti-CD73 (TY/11.8), anti-Tim-3 (RMT3-23), and anti-OX40 (OX-86), for 20 min in the dark. Antibodies were obtained from BioLegend (San Diego, CA, USA), BD Biosciences (Franklin Lakes, NJ, USA), and Thermo Fisher Scientific (Waltham, MA, USA). To wash out unbound antibodies, the cells were washed again with PBS. The cells were then ready for intracellular staining via an intracellular staining kit (Thermo Fisher Scientific, Waltham, MA, USA). For intracellular staining, the cells were first mixed with 500–1000 μL Fix/Perm reagent and incubated for 30–45 min at 6 °C. This process fixes the surface staining and permeabilizes the cell membrane for the penetration of intracellular antibodies. The reagent was then washed off with 2 mL of the Perm Buffer by centrifugation at 6 °C. The intracellular antibody FOXP3 (FJK-16s) was then added. The intracellular staining was incubated for 30–45 min at 6 °C. The intracellular staining was washed off with buffer as in the previous step and could be stored at 6 °C for up to 7 days until the time of measurement. For measuring compensation controls, single-stained BD^™^CompBeads (Anti-Hamster Ig, k/Negative Control Compensation Particle Set, BD Biosciences, Franklin Lakes, NJ, USA) were used. For live/dead (Zombie) compensation, compensation beads stained with anti-CD19 (APC Cy-7, BioLegend, San Diego, CA, USA) were used. All samples were acquired on a BD FACSymphony^™^ A3 with BD FACSDiva software version 8 (BD Biosciences, Franklin Lakes, NJ, USA). SPICE analysis was performed with the software SPICE (Version 6.1) [19].

### 4.3. Ex Vivo Cytotoxicity Assay

Syngenic T cell ex vivo cytotoxicity assays were performed by using murine CD3^+^ cells from leukemic mice (n = 3) or HD mice (n = 3) as effector cells and the C1498 AML cell line as target AML cells. The CD3^+^ cells were collected via a MojoSort^TM^ magnetic beat T-cell isolation kit (BioLegend, San Diego, CA, USA) according to the manufacturer’s protocol from freshly crushed murine spleens of either leukemic or HD mice. T cells were then cocultured with the C1498 cells, which were labeled with CellTracker^™^ (CT) green CMFDA (Invitrogen Thermo Fisher, Waltham, MA, USA) according to the manufacturer’s instructions, in a 6:1 effector:target ratio. Coculture was plated in a 96-well plate (1 × 10^6^ cells/mL) in RPMI culture medium and incubated with a TIGIT-blocking antibody for 24 h at 37 °C and 5% CO_2_. Each well of the 96-well plate contained a 200 µL volume in total, including the blocking antibodies. For experiments with a single blockade of TIGIT, cocultured cells were incubated with 25 µg/mL anti-mouse TIGIT antibody (anti-mouse TIGIT clone F132-1B4, kindly provided by Prof. Vijay Kuchroo, Harvard Medical School) or mouse IgG1 isotype control (BioLegend, San Diego, CA, USA). Experiments were performed in triplicates and repeated three times with the same results. Bars display the mean ± SD. After 24 h of incubation, the cells of each well were collected and washed with PBS and FcR blocking was performed. After a second PBS washing step, cells were stained with 7-Aminoactinomycin D (7-AAD) Viability Staining Solution (BioLegend, San Diego, CA, USA) according to the manufacturer’s protocol. After an incubation time of 10 min at room temperature, the samples were subsequently analyzed on a BD FACSCanto with BD FACSDiva software version 8 (BD Biosciences, Franklin Lakes, NJ, USA).

The frequency of AML cell lysis was determined via positivity of 7-AAD and CT green using flow cytometry.

### 4.4. Cell Lines

The AML cell line C1498 was purchased from the American Type Culture Collection, and cells were cultured in DMEM + GlutaMAX^TM^ (Gibco, Thermo Fisher Scientific, Waltham, MA, USA) and supplemented with 10% fetal bovine serum (FBS superior, Sigma-Aldrich, St. Louis, MO, USA). Murine CD3^+^ cells were cultivated in RPMI1640 + GlutaMAX^TM^ (Gibco, Thermo Fisher Scientific, Waltham, MA, USA) and supplemented with 10% fetal bovine serum. Cell cultures were incubated at 37 °C and 5% CO_2_.

### 4.5. Statistical Analysis

All flow cytometric data were analyzed using FlowJo version 10.4 and 10.5.2 software (BD Life Sciences, FlowJo, LCC, Ashland, OR, USA). Statistical analyses were carried out using Prism 8.0 (GraphPad Software, San Diego, CA, USA). All groups were tested for normal distribution with the Kolmogorov–Smirnov test and the Shapiro–Wilk test. The normally distributed data were analyzed with the ordinary one-way ANOVA. Non-normally distributed data were analyzed via the Kruskal–Wallis tests for more than two groups. The normally distributed data from the cytotoxicity assay were analyzed by the unpaired *t* test for two unpaired groups. Frequencies in the text are described as medians unless stated otherwise (as indicated in the figure legend). *p*-Values smaller than 0.05 were considered significant, with *, **, ***, and **** indicate *p*-values between 0.01 and 0.05, 0.001 and 0.01, 0.0001 and 0.001, and <0.0001 respectively.

## 5. Conclusions

In conclusion, the study shows that in our murine C57BL/6 model, reg CD4^+^ T cells represent the dominant T-cell fraction in leukemic spleens. Moreover, these reg CD4^+^ T cells contained a more mature differentiation status and an aberrant expression of co-regulatory checkpoint molecules. Further functional studies should be conducted to investigate whether blockade of TIGIT alone or in combination with the blockade of other checkpoint molecules can enhance antileukemic cytotoxicity.

## Data Availability

The datasets used and/or analyzed during the current study are available from the corresponding authors upon reasonable request (julius.krueger@stud.uke.uni-hamburg.de).

## Supplementary Materials

The following supporting information can be downloaded at: https://www.mdpi.com/article/10.3390/ijms252111412/s1.
