# Supplementary material for: Murine Regulatory CD4+ T Cells Are Increased in Leukemic Spleens and Show High Co-Expression of Co-Regulatory Molecules CD39, CD73, PD1, and TIGIT
Source: Int J Mol Sci. 2024 Oct 24;25(21):11412. doi: 10.3390/ijms252111412 (PMC11546357; doi:10.3390/ijms252111412)
Supplement: Supplementary file 1 [file ijms-25-11412-s001.zip › ijms-3211584-supplementary.pdf]

Supplementary Figure S1

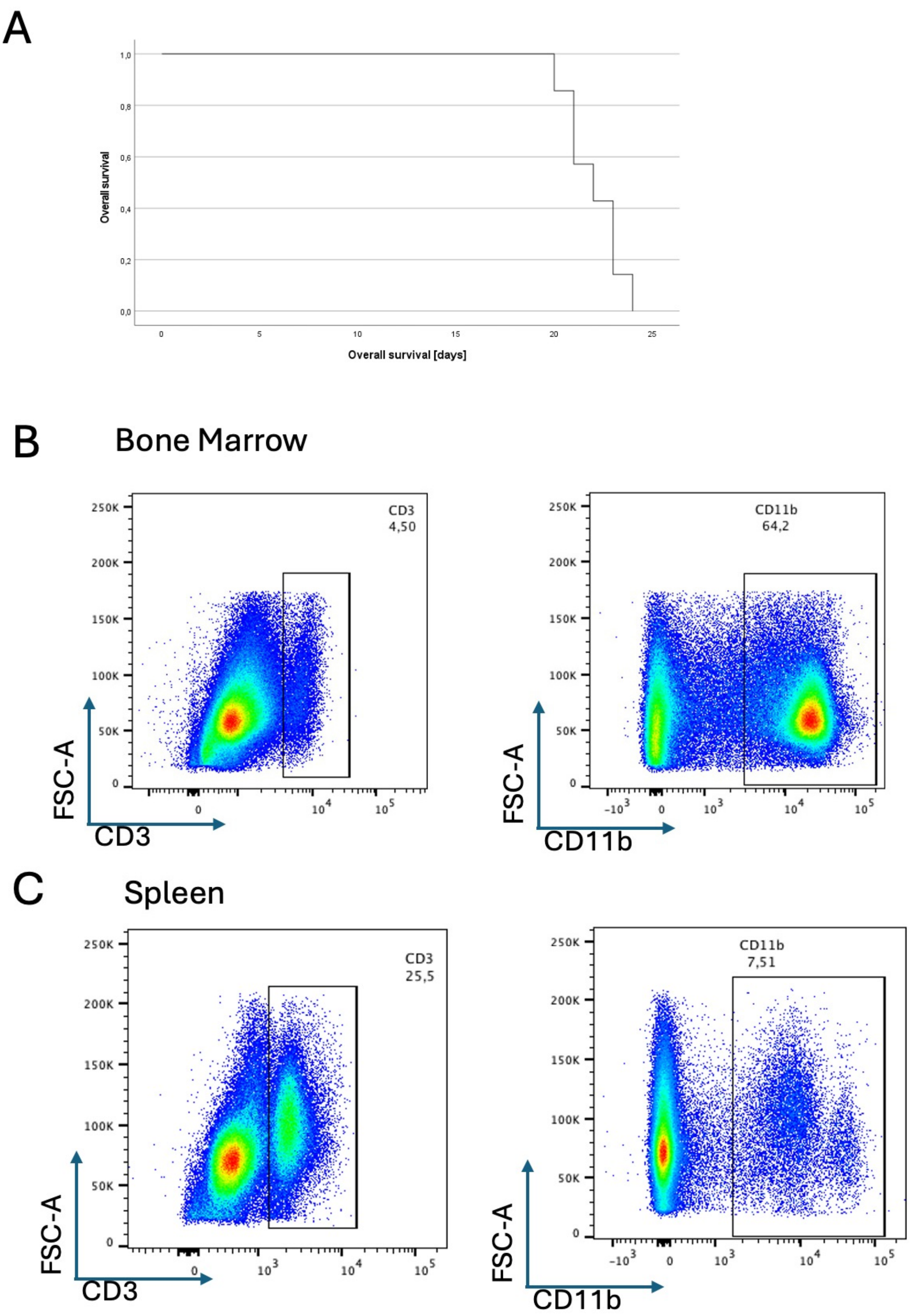

Supplemental Figure 1. Survival curve of n=7 C57 BL/6 mice after injection of 10^6 C1498 AML cells (**A**). Exemplary staining of different frequencies of $CD3^+$ cells and $CD11b^+$ cells in either (**B**) bone marrow cells or (**C**) spleen cells

Supplementary Figure S2

A

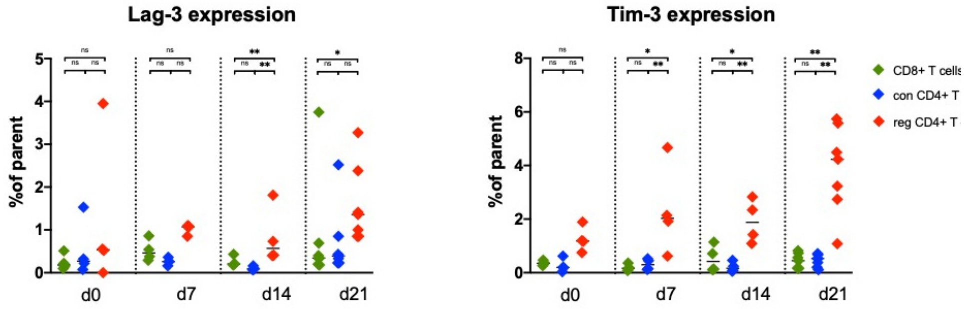

B

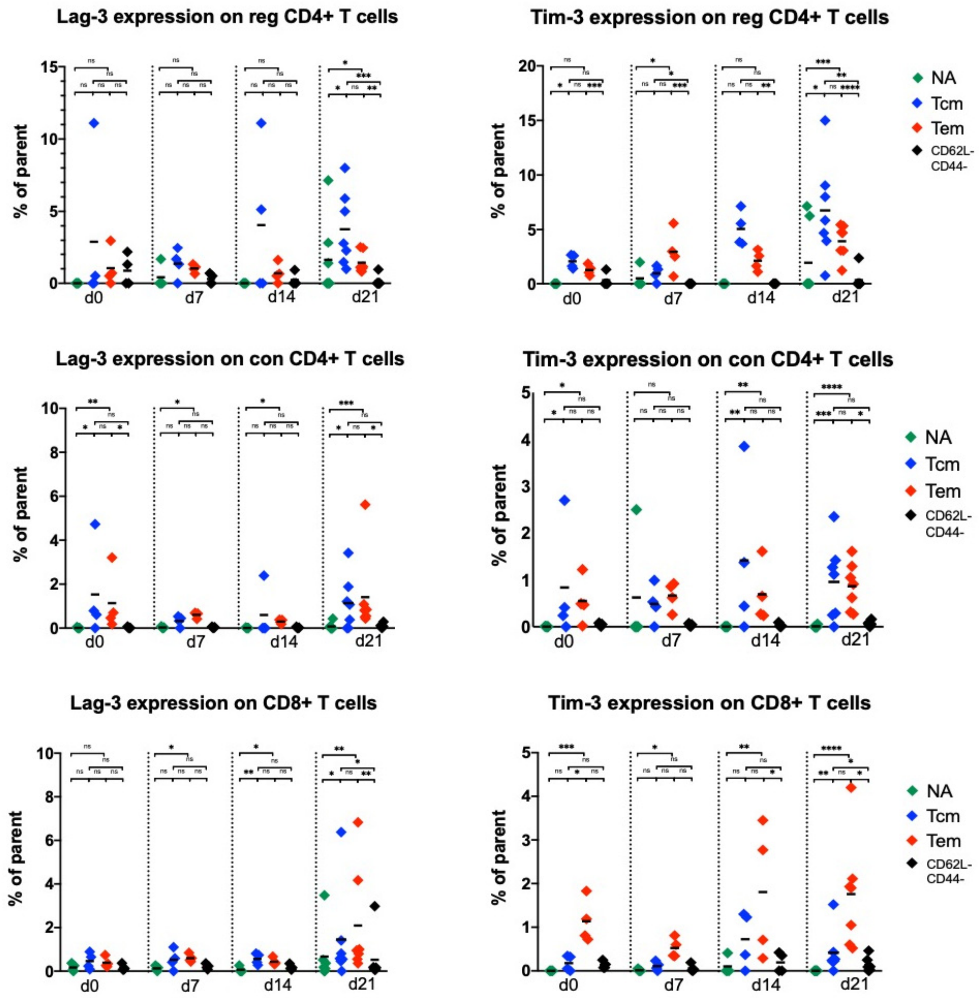

Supplementary Figure S2 (**A**) Summary Data of IC expression on CD4^{+} T cells, CD8^{+} T cells and reg CD4^{+} T cells. (**B**) Summary data of immune checkpoint expression on T cell subgroups. Frequencies are displayed with the median. P values were obtained by the Kruskal-Wallis test. *P<0.05, **P<0.01, ***P<0.001, ****P<0.0001.

Supplementary Figure S3

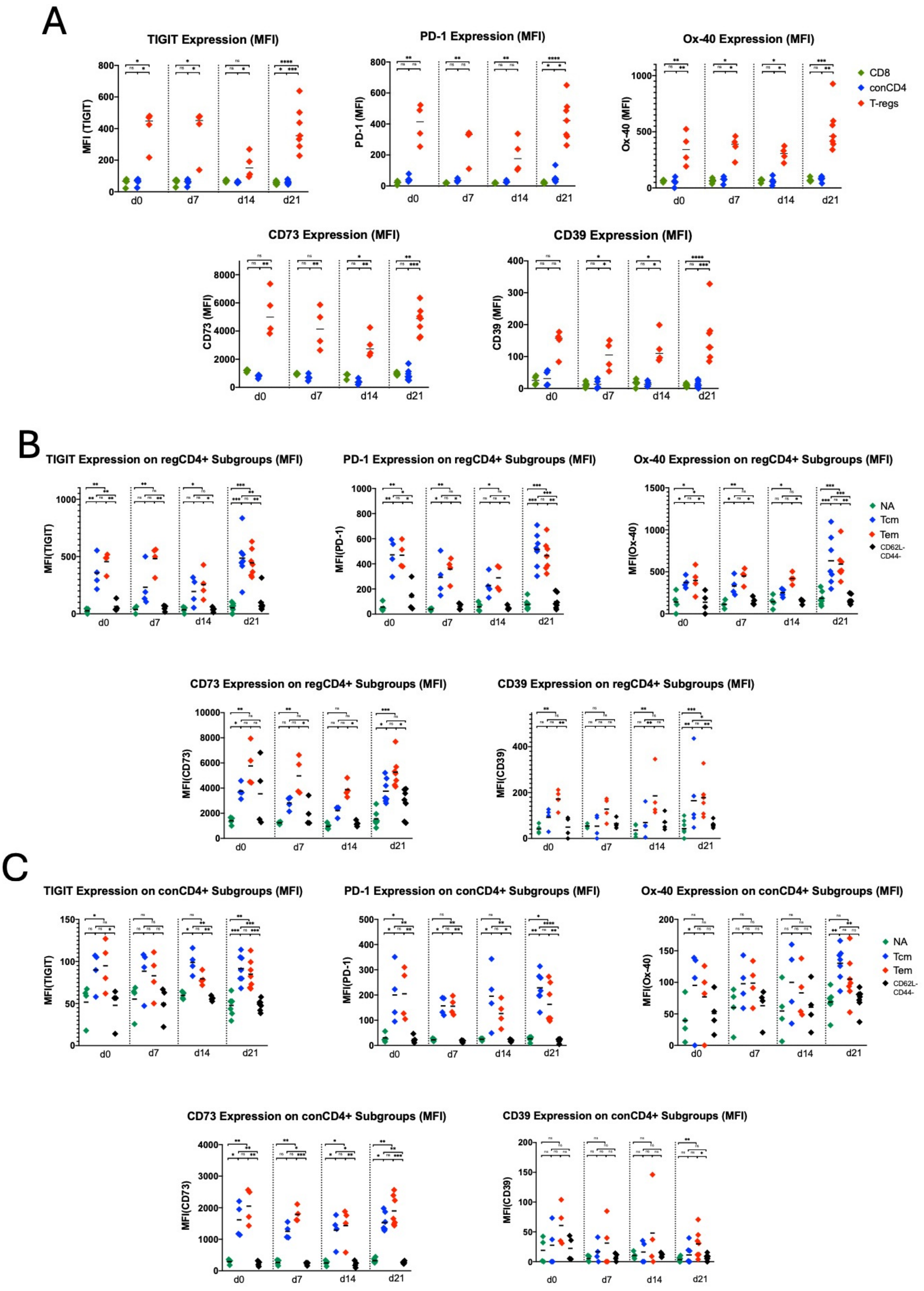

Supplementary Figure S3 (**A**) Summary Data of IC expression on CD4+ T cells, CD8+ T cells and reg CD4+ T cells. (**B**) Summary data of immune checkpoint expression on T cell subgroups. Frequencies are displayed as the Median Flourescence Intensity. P values were obtained by the Kruskal-Wallis test. *P<0.05, **P<0.01, ***P<0.001, ****P<0.0001.

Supplementary Figure S4

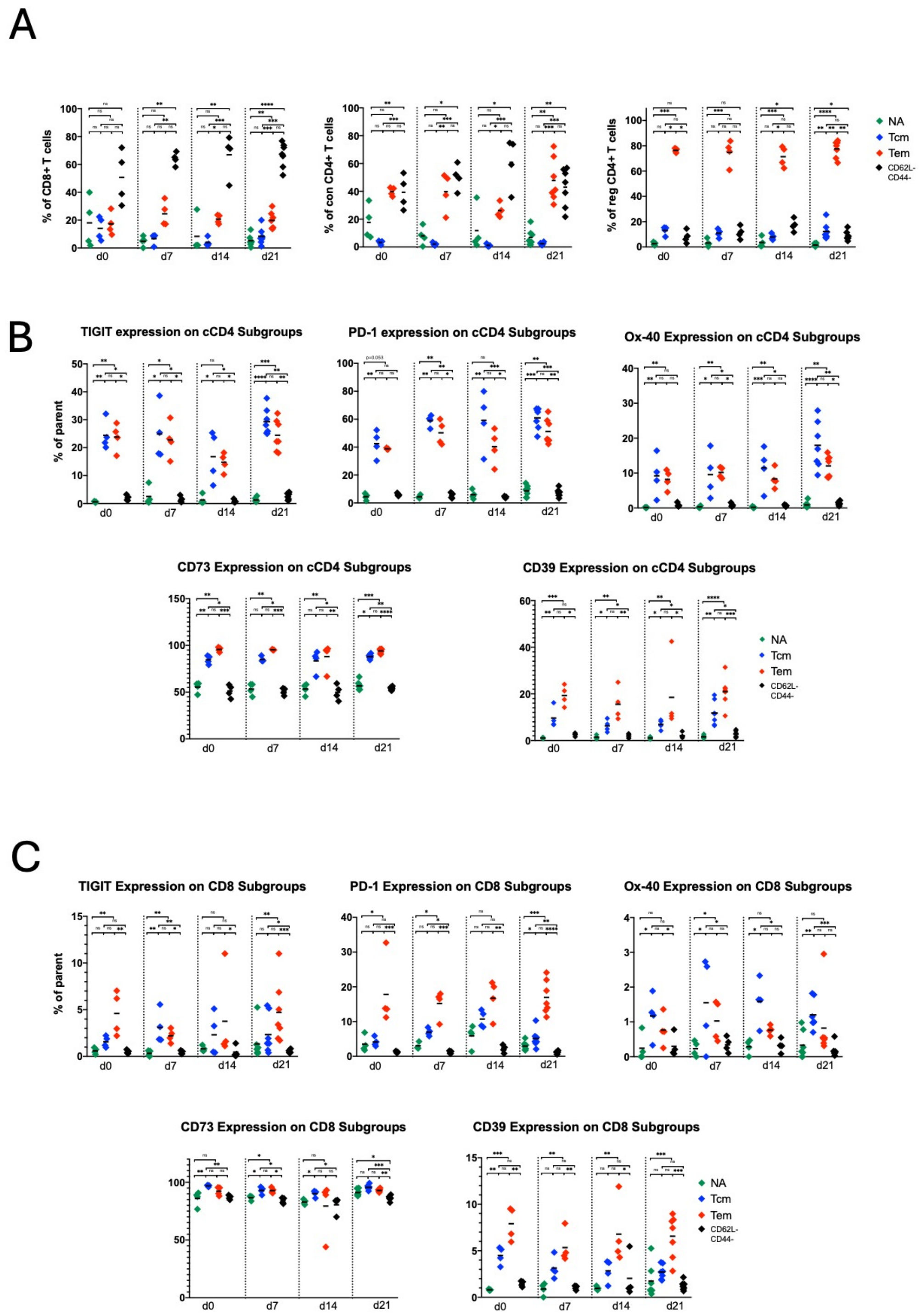

Supplementary Figure S4 (**A**) Summary Data of the distribution of T cell subgroups NA = $CD62L^{+}CD44^{-}$, Tem = $CD62L^{-}CD44^{+}$, Tcm= $CD62L^{+}CD44^{+}$ and DN (double negative) = $CD62L^{-}CD44^{-}$ subpopulations (**B+C**) Summary data of immune checkpoint expression on T cell subgroups. Frequencies are displayed as the Median Flourescence Intensity. P values were obtained by the Kruskal-Wallis test. *P<0.05, **P<0.01, ***P<0.001, ****P<0.0001.
